# Dysregulated YY1/PRMT5 axis promotes the progression and metastasis of laryngeal cancer by targeting Hippo pathway

**DOI:** 10.1111/jcmm.16156

**Published:** 2020-12-07

**Authors:** Nan Wang, Di Wu, Qian Long, Yue Yan, Xiaoqi Chen, Zheng Zhao, Honghong Yan, Xinrui Zhang, Meilan Xu, Wuguo Deng, Xuekui Liu

**Affiliations:** ^1^ School of Life Sciences Jiaying University Meizhou China; ^2^ Sun Yat‐sen University Cancer Center State Key Laboratory of Oncology in South China Collaborative Innovation Center of Cancer Medicine Guangzhou China

**Keywords:** laryngeal cancer, LATS2, metastasis, PRMT5, YY1

## Abstract

Metastases lead to high mortality in laryngeal cancer, but the regulation of its underlying mechanisms remains elusive. We identified Protein arginine methyltransferase 5 (PRMT5) was significantly up‐regulated in laryngeal cancer tissues, which predicts poor patient prognosis. Functional assays demonstrated that PRMT5 overexpression promoted the invasive capacity and lymph node metastasis in vitro and in vivo. Mechanistic experiments suggested that LATS2 was a downstream target of PRMT5. PRMT5 inhibition increased the expression of LATS2 and YAP phosphorylation in laryngeal cancer cells, thereby promoting laryngeal cancer metastasis. Furthermore, informatics and experimental data confirmed that *PRMT5* gene was transcriptionally activated by YY1. Collectively, our results unravelled the important role of PRMT5 in laryngeal cancer tumorigenesis and metastasis. The dysregulation YY1/PRMT5/LATS2/YAP axis may contribute to laryngeal cancer progression; thus, PRMT5 may be a potential therapeutic strategy for patients with laryngeal cancer.

## 1 INTRODUCTION

Laryngeal cancer, the leading cause of death in otolaryngology worldwide, is characterized by a high rate of local invasion and lymph node metastasis.^1^ The incidence of laryngeal cancer is increasing annually, with about 177,422 new cases worldwide.^2^ Despite recent advancements in therapeutic strategies, the overall 5‐year survival rate of lymph node metastatic patients remains poor.^3^ Until now, no effective diagnostic biomarkers are available for patients with lymph node metastasis. Thus, it is urgent to explore the understanding mechanisms of laryngeal cancer initiation and metastasis as well as search for effective diagnostic biomarkers, drug targets to improve the survival of advanced diseases.

PRMT5 is a major type II arginine methyltransferase and regulates gene expression by symmetric dimethylation of histone and non‐histone proteins. Accumulating evidence shows that PRMT5 is overexpressed in several human cancers and correlates with poor prognosis.^4^, ^5^, ^6^, ^7^, ^8^ PRMT5 has been reported to be complexed with the WD‐repeat protein MEP50, and elevated PRMT5/MEP50 has been found in many solid cancers and correlated with enhanced tumour growth and worse prognosis.^9^, ^10^ Furthermore, PRMT5 may function as an oncogene by epigenetic repression of tumour suppressor genes or by post‐translational modification of signalling molecules.^11^, ^12^ In acute myeloid leukaemia, PRMT5 was recruited to the snail‐AJUBA protein complex to participate in the transcriptional silencing of E‐cadherin leading to invasiveness.^13^ Moreover, one of the most recent report linked PRMT5 to epithelial‐mesenchymal transition (EMT) during oncogenesis and progression of oral squamous cell carcinoma.^14^ However, it remains unclear whether PRMT5 has overt oncogenic function in the context of laryngeal cancer cells. As an emerging cancer treatment target, PRMT5 inhibitors have been developed and some of them is ongoing clinical study.^15^, ^16^, ^17^ However, these PRMT5 enzyme activity‐based inhibitors due to lack of substrate specificity limited its application. Therefore, it is important to uncover the oncogenic role of PRMT5 and identify novel more specific inhibitor in anti‐laryngeal cancer treatment.

Transcription factors (TFs) play a leading role in cancer initiation.^18^, ^19^ Dysregulated TFs in multiple cancers have been suggested as appropriate targets for the development of anticancer drugs.^20^ The transcription factor Yin Yang 1 (YY1) is the first identified member of the Yin Yang family, a GLI‐Krüppel zinc finger protein family that contains four highly conserved C2H2 zinc finger domains.^21^ YY1 is a multifunctional factor, can act as an activator or a repressor depending on the context and the recruitment cofactors in gene expression..^22^, ^23^ YY1 is overexpressed in various cancers and facilitated tumorigenesis and angiogenesis.^24^, ^25^, ^26^ Recent studies have indicated that YY1 is inversely correlated with E‐cadherin and play a pivotal role in EMT and hepatocellular cancer metastasis.^27^ On the other hand, YY1 has been reported to be a tumour suppressor in pancreatic cancer, in which its highly expression inhibited cell invasion and metastasis.^28^ Moreover, YY1 acts as an inhibitor by inhibiting proliferation of breast cancer and glioblastoma cells.^29^ However, the role of YY1 in the progression of laryngeal carcinogenesis has not been explored before.

The Hippo pathway is highly conserved and has multiple biological functions in regulating organ size, tissue homeostasis and the regeneration of tissues.^30^, ^31^ Typically, LATS, YAP/TAZ, MOB1 and SAV1 are the core components of Hippo pathway.^32^, ^33^ Yes associated protein 1 (YAP) and Transcriptional co‐activator with PDZ‐binding motif (TAZ) are the major effectors of this pathway, which was reported to be involved in the invasiveness and migration of breast cancer.^34^ In addition, YAP was considered as a prognostic factor and associated with lymph node metastasis in HNSC (Head and neck squamous cell carcinoma).^35^ However, how these factors affect laryngeal cancer progression through Hippo pathway have not yet been fully elucidated.

In the present study, we investigated the role of PRMT5 in regulating laryngeal cancer progression and metastasis. We found that PRMT5 was transcriptionally activated by YY1 and triggered proliferation and invasion via inhibiting LATS2 and phos‐YAP of Hippo signalling pathway in laryngeal cancer cells. Our results demonstrate that inhibition of PRMT5 could represent an promising therapeutic stragety for the treatment of laryngeal cancer.

## 2 MATERIALS AND METHODS

### 2.1 Clinical specimens and cell lines

Fresh tissues from 19 laryngeal cancer specimens were collected for determination of mRNA and protein levels of PRMT5 from the Sun Yat‐sen University Cancer Center of Sun Yat‐sen University. Informed consent was obtained from the patients before the study began, none of whom had received chemotherapy or radiotherapy prior to surgery. Another archival formalin‐fixed paraffin‐embedded tissues with primary and lymph node metastatic tumour and normal tissues from 80 patients, who underwent laryngectomy and/or metastasectomy, were collected from January 2013 to August 2016 in Medical College of Jiaying University. None of the patients received radiotherapy or/and chemotherapy before surgery. The clinicopathological information was retrieved and summarized. Laryngeal cancer patients gene expression was obtained from the TCGA Data Portal website (https://portal.gdc.cancer.gov/projects/). This study was conducted according to the Declaration of Helsinki.

Human cells Tu212, Tu686 and 293T were purchased from Guangzhou Juyan Biological Technology (Guangzhou, China). CAL‐27, HOK and SCC‐15 cells were obtained from Beina Biological Technology (Beijing, China). All cell lines were validated by STR (short tandem repeat) assays and cultured in RPMI 1640 (Gibco, USA) supplemented with 10% FBS (Gbico, USA) and 1% penicillin‐streptomycin solution (Invitrogen, USA) in a humidified atmosphere of 5% CO2 at 37°C.

### 2.2 Lentivirus infection, RNA interference and plasmid transfection

PRMT5 short hairpin RNA (shRNA) and overexpression lentivirus were cloned into the pCDH‐CMV‐MCS‐EF1‐Puro or pEZ‐Lv217.1‐Luc‐Puro vectors, respectively. PRMT5‐targeting shRNAs oligonucleotide sequences are listed in Table S1. The recombinant lentivirus was produced by co‐transfecting 293T cells with PRMT5‐shRNA and two lentivirus packaging plasmids (psPAX2 and pMD2.G) by using Lipofectamine 3000 (Invitrogen, Thermo Fisher Scientific). The virus‐containing supernatant was harvested at 72 hours after transfection. For stable overexpression of PRMT5, pEZ‐Lv217.1‐Luc‐Puro plasmid and two lentivirus packaging plasmids were used. Stable cell lines were selected using 1 μg/mL puromycin dihydrochloride (Invitrogen) for 14 days. The efficiency of PRMT5 suppression and overexpression were confirmed by Western blot and qRT‐PCR. siRNA oligonucleotides targeting PRMT5, YY1 and negative control siRNA were purchased from Ribobio (Guangzhou, China). siRNA transfections were used Lipofectamine RNAimax (Life Technologies) according to the manufacturer's instructions. After a 48 hours period of transfection, the cells were collected for subsequent experimentation. The plasmids were transfected in strict accordance with the instructions of lipofectamine 3000. All siRNAs sequences used in the present study were listed in Table S2.

### 2.3 Quantitative real‐time PCR

The SYBR green qPCR Master Kit (Thermo Fisher Scientific) was used for PCR amplification. Total RNA was isolated and reverse‐transcribed according to the protocol as previously described.^36^ RNA concentration was assessed by Nano Drop 2000 (Thermo Fisher Scientific). The relative expression level of mRNAs was determined by 7900HT Fast Real‐Time PCR system (Applied Biosystems). Primers used in the study were listed in Table S3. The experiments were repeated three times independently.

### 2.4 Western blot analysis

Western blot analysis was performed as previously described.^36^ The antibodies used in the present study were as follows: PRMT5 (1:1000), Vimentin (1:1000), Snail (1:1000), LATS2 (1:1000), p‐YAP (S127) (1:1000), E‐cadherin (1:1000) and GAPDH (1:1000) were purchased from Cell Signaling Technology. YY1 (1:300), MMP9 (1:1000) and Cyclin D1 (1:500) were from Proteintech. YAP (1:2000) was provided by Abcam. Signals were detected using Chemi Dox XRS instrument plus Image lab software (BIO‐RAD).

### 2.5 Immunohistochemistry

Immunohistochemistry (IHC) was performed to detect PRMT5, E‐cadherin, Vimentin, MMP9, YY1 and Ki‐67 expression. Paraffin‐embedded tumour tissues were deparaffinization and antigen‐retrieval, sectioned and stained with PRMT5 (1:800, CST), E‐cadherin (1:100, Servicebio), Vimentin (1:150, Servicebio), MMP9 (1:200, Servicebio), YY1 (1:50, Proteintech) antibodies at 4 ℃ overnight, respectively. Then, after washed three times with PBS, the sections were incubated with secondary antibody at room temperature for 2 hours. The immunostaining was visualized by diaminobenzidine staining solution (Bioss, Beijing, China). The expression of PRMT5 was evaluated and quantified by two independent pathologists according to a staining scoring system. Five microscopic visual fields (X200) were randomly selected from each slice to ensure representation of the sample. Photographs were taken with Olympus BX53 Microscope system. The median IHC scores >4 were classified as high expression, and scores of 0‐4 were classified as low expression.

### 2.6 RNA‐seq analysis

Total RNAs were extracted from laryngeal cancer cells using TRIzol reagent (Invitrogen). cDNA library construction and high‐throughput sequencing results analysis were performed by Shanghai Sinotech Genomics. The differentially expressed genes were screened by the threshold of 1.5‐fold change and *p* value that was more than 0.05. Pathway analysis was used to elucidate the significant pathway according to the KEGG database.

### 2.7 Cell proliferation and cell cycle assays

The CCK8 assay was performed to detect cell proliferation. For transient transfection experiments, 1 × 10^3^/well cells were seeded in 96‐well plates at 37℃ for 24 hours. The proliferation absorbance at 450 nm was measured using a Spark® multimode microplate reader (Tecan, Männedorf). Experiments were repeated three times. For cell cycle assay were performed with propidium iodide‐stained laryngeal cancer cells by flow cytometry (BD FACS Canto II, BD Biosciences, NJ, USA) and analysed using the Modfit software.

### 2.8 In vitro migration and invasion assays

For in vitro invasion and migration assays, 5 × 10^4^ cells were suspended in serum‐free medium and seeded into the upper chambers (24‐well insert; pore size, 8 μm; Corning) with or without precoated matrigel (BD Biosciences). 500 μL RPMI‐1640 medium containing 20% foetal bovine serum (FBS) was added to the lower chamber of each well. After 36 hours incubation, cells in the upper chamber were removed using a cotton swab and cells located on the underside of the membrane were fixed with 4% paraformaldehyde and stained with 0.1% crystal violet solution (Beyotime Biotechnology). Five randomly selected fields at ×200 magnification were imaged and counted. Three independent experiments were carried out for this.

### 2.9 Luciferase reporter assay

A dual‐luciferase assay was performed to confirm the impact of YY1 on *PRMT5* promoter activity. Briefly, cells on a 24 well plate were co‐transfected with the firefly luciferase reporter (50 ng) along with the Renilla luciferase reporter (Promega) (20 ng) for 24 hours using Lipofectamine 3000 according to the protocol provided by manufacturers. The luciferase activity was measured in cellular extracts using a Dual‐luciferase Reporter Assay Kit (Promega). The luciferase activity of each well was normalized to Renilla luciferase activity. The experiment was repeated in triplicate.

### 2.10 In vivo metastasis and tumorigenesis assay

For lymphatic metastasis, 2 × 10^6^ cells Tu686 cells with or without PRMT5 suppression were inoculated into the right lower footpads of BALB/c‐nu mice (4‐5 weeks of age). The tumour growth and metastasis were monitored and imaged by injecting D‐luciferin substrate (Promega) and Bruker In‐Vivo FX PRO System (Bruker company, Germany) every one week after injection. Six weeks later, the mice were killed and the tumours were embedded in paraffin for later detection. To evaluate the effect of PRMT5 on tumorigenesis, BALB/c‐nu mice were subcutaneously injected 1 × 10^7^ cells into the right flank. The tumour size was measured every 3 days, and tumour volume was calculated as ( width^2^ × length)/2. On day 30, the mice were killed and tumour tissues were collected, fixed in 4% paraformaldehyde and embedded in paraffin. The animal experiments were approved by the Institute Animal Care and Use Committee of Cancer center of Sun Yat‐sen University Cancer Center according to the protocols.

### 2.11 ChIP assay

Cells were grown to 90% confluence and then treated with 1% formaldehyde to cross‐link proteins to DNA. The crosslinking, immunoprecipitation, washing, elution, reverse crosslinking and proteinases K treatment were performed according to the manufacturer's directions described in the Simple ChIP Enzymatic Chromatin IP Kit #9003 (Cell Signaling Technology, Inc). Purified immunoprecipitated DNA was used for real‐time qPCR. Primers for ChIP PCR were as follows: sense CGAGACCAGCCTGACCAACA, antisense CTTCCAGCAACCAATCAGAT.

### 2.12 Statistical analysis

The data are presented as the mean ± SD from three independent experiments. The GraphPad Prism 6 software (San Diego, CA, USA) was used for statistical analysis. Comparisons between two independent groups were analysed with two‐tailed Student's t test. Statistically significance was assigned at *P < .05, **P < .01 and ***P < .001.

## 3 RESULTS

### 3.1 PRMT5 is highly expressed and predicts poor prognosis in laryngeal cancer

**FIGURE 1:**
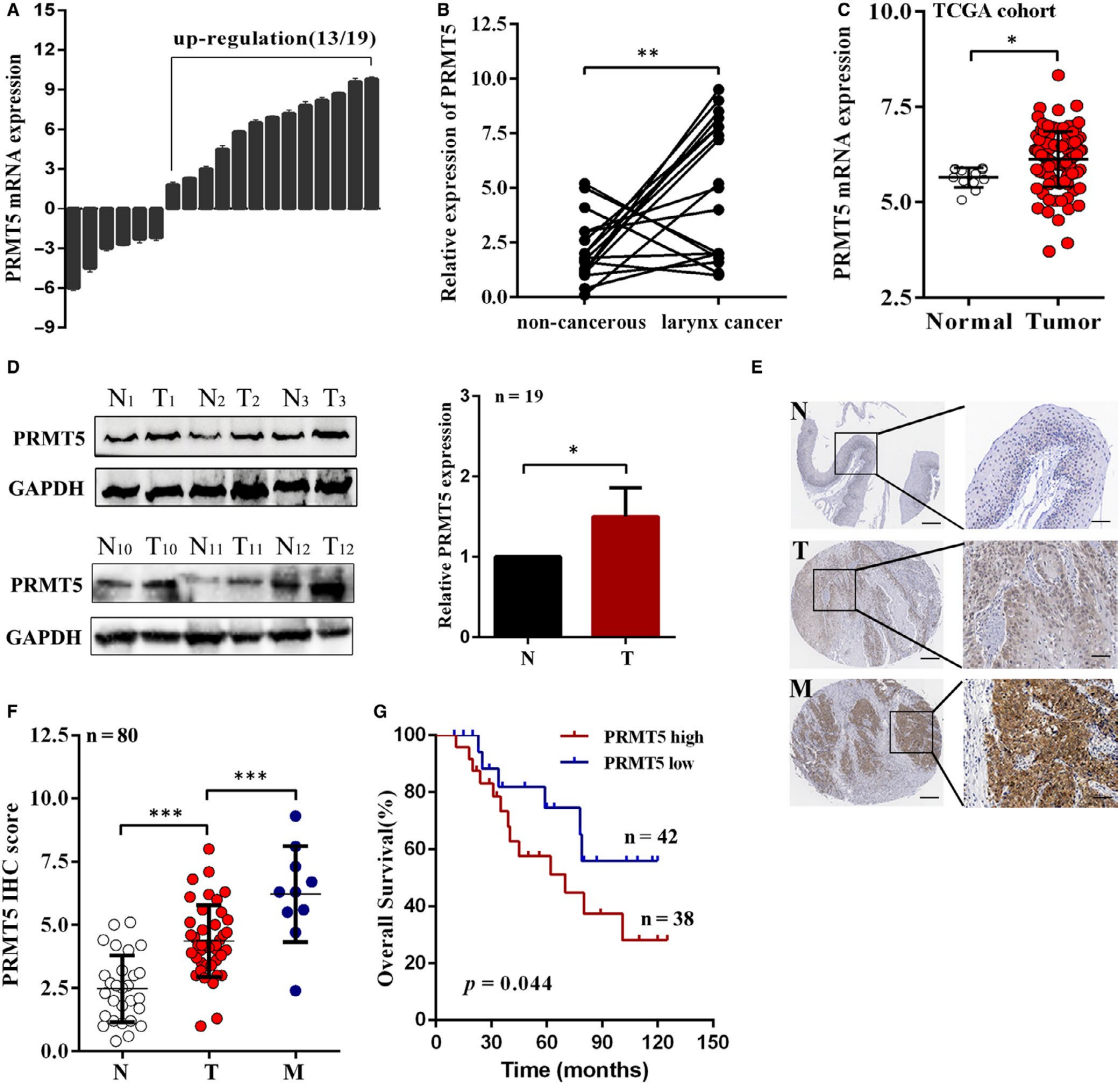
PRMT5 is highly expressed and predicts poor prognosis in laryngeal cancer. A, Real‐time PCR analysis of PRMT5 mRNA in 19 paired laryngeal cancer tissue and normal tissue samples. B, As analysed by RT‐PCR, PRMT5 expression in laryngeal cancer tissues was significantly higher than that in the corresponding non‐cancerous tissues. C, The up‐regulation of PRMT5 mRNA was found in TCGA cohort. D, The representative images of PRMT5 protein expression in laryngeal cancer tissues were examined by Western blot. E, Representative IHC images showing the expression of PRMT5 in non‐tumorous, laryngeal cancer and metastatic tissues. F, IHC scores showed the expression of PRMT5 in non‐tumorous, laryngeal cancer and metastatic tissues. G, Kaplan‐Meier analysis of the correlation between PRMT5 expression and overall survival showed that patients with higher levels of PRMT5 had shorter overall survival. n = 80. *P < .05, **P < .01, ***P < .001

To understand the expression profile of PRMT5 in laryngeal cancer, we first assessed the expression of PRMT5 in fresh laryngeal cancer tissues and paired non‐cancerous tissues. The mRNA level of PRMT5 was frequently up‐regulated in 13 of the 19 paired tissues (Figure 1A,B). The up‐regulation of PRMT5 in laryngeal cancer was validated in a 123‐case cohort from The Cancer Genome Atlas (TCGA) (Figure 1C). Consistently, the protein expression of PRMT5 was higher in laryngeal cancer specimens (Figure 1D, Figure S1). We further performed IHC of PRMT5 on a cohort consisting 80‐case tissues and found PRMT5 was overexpressed in primary and metastatic tumour tissues compared with the normal tissues (Figure 1E, F). These data suggest that PRMT5 expression was gradually augmented from primary tumour to metastatic tumours. Furthermore, the patients were classified into two groups (PRMT5 ^high^ and PRMT5 ^low^) according to the median IHC score of PRMT5 expression (4.0). The correlations between the clinicopathologic features of laryngeal cancer patients and PRMT5 were summarized in supplementary Table S4, which revealed that PRMT5 expression is positively correlated with advanced tumour stage and lymph node metastasis status. More importantly, Kaplan‐Meier survival analysis showed that the laryngeal patients with high levels of PRMT5 had shorter overall survival compare those with low levels of PRMT5 (Figure 1G). These observations indicated that PRMT5 is up‐regulated in laryngeal cancer tissues and may be involved in laryngeal cancer progression.

### 3.2 PRMT5 knockdown inhibits laryngeal cancer cell invasion and lymph node metastasis in vitro and in vivo

**FIGURE 2:**
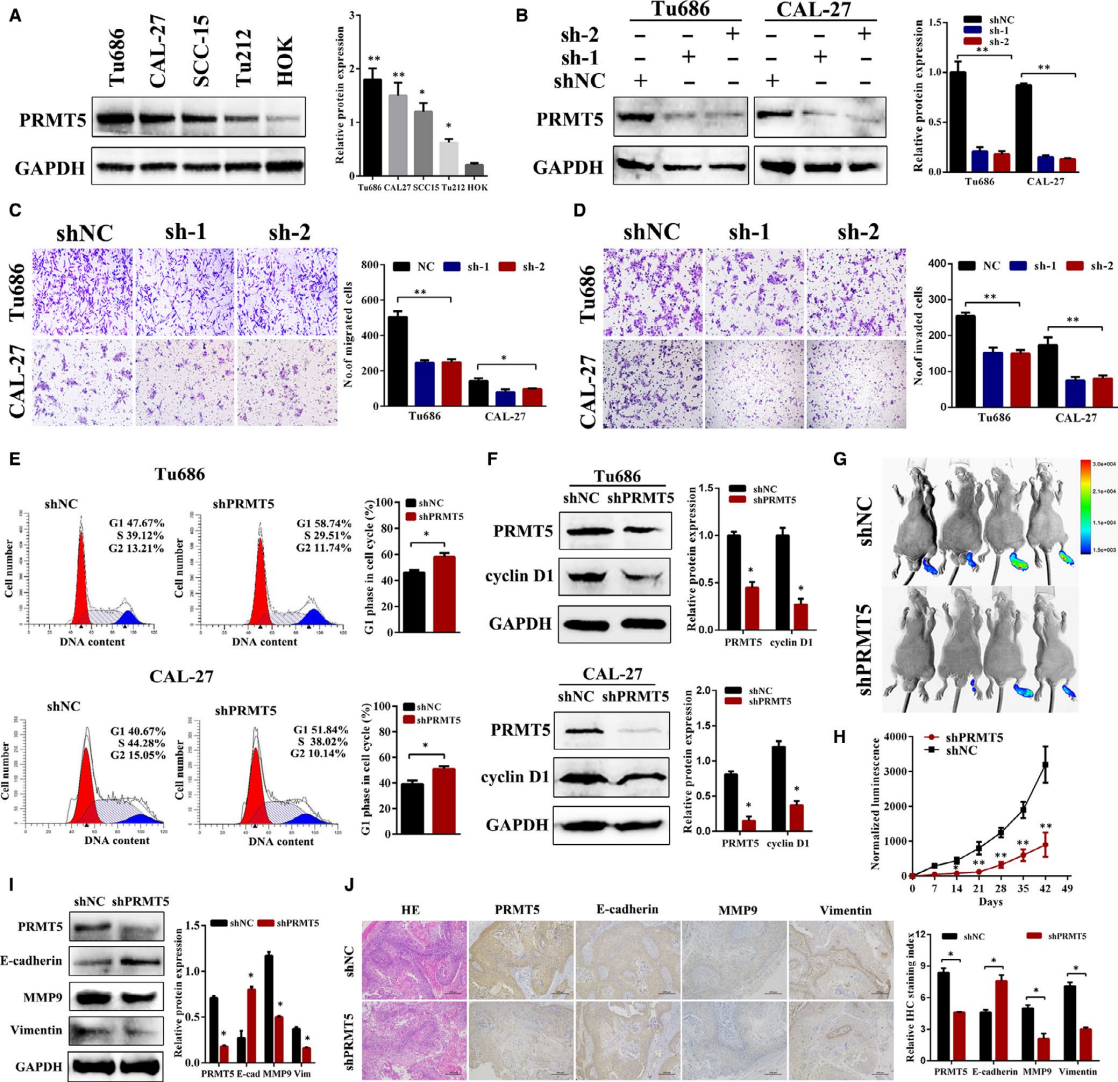
Knockdown of PRMT5 by shRNAs suppresses laryngeal cancer metastasis. A, Western blot analysis showing the expression levels of PRMT5 in laryngeal cancer cell lines and HOK. B, The knockdown efficiency of shPRMT5 in Tu686 and CAL‐27 cells by Western blot. C‐D, Knockdown of PRMT5 by two different shRNAs suppressed cell migration and invasion in Tu686 and CAL‐27 cells. Representative images and quantification analyses were shown (mean ± SD, n = 3, *P < .05. **P < .01). E, Representative images shown that knockdown of PRMT5 increased the G1 fraction, as detected by flow cytometry. All the experiments were repeated three times. F, Western blot analysis revealed that shPRMT5 suppressed cyclin D1 expression in Tu686 and CAL‐27 cells. G, Bioluminescence images for mice in the indicated groups. H, Relative changes in the luminescence of xenograft tumours formed by shPRMT5 and control shNC. I, Western blot analysis of PRMT5, E‐cadherin, MMP9 and Vimentin in tumour xenograft tissues. J, Representative HE and immunohistochemistry staining of PRMT5, E‐cadherin, MMP9 and Vimentin in the indicated xenografts. Scale bar, 100 μm. The staining intensity are presented as their mean ± SD. Statistical analysis was performed with the Student's t test. *P < .05, **P < .01

To ascertain the function of PRMT5 in laryngeal cancer, we performed Western blot analysis in a panel of cell lines, which included CAL‐27, Tu212, Tu686, SCC‐15 and human oral keratinocytes (HOK). As shown in Figure 2A, PRMT5 was highly expressed in laryngeal cancer cell lines. Next, we generated two independent short hairpin RNAs against *PRMT5*, which could efficiently reduce the endogenous expression of *PRMT5* in Tu686 and CAL‐27 of laryngeal cancer cells (Figure 2B). As illustrated in Figure 2C, D, knockdown of *PRMT5* significantly inhibited cell migration and invasion in vitro. As PRMT5 depletion using two separate shRNAs (sh‐1 and sh‐2) achieved the same reduction in Figure 2B‐D. We chose to use sh‐1 and label as shPRMT5 in this study. As illustrated in Figure 2E, flow cytometry analysis showed that shPRMT5 inhibited cell growth through inducing cell cycle arrest at G1 phase in the laryngeal cancer cell lines. Western blot analysis revealed that shPRMT5 suppressed the G1‐S transition promoter cyclin D1, confirming that shPRMT5 blocked the cell cycle at the G1/S checkpoint (Figure 2F).

To study the effect of PRMT5 knockdown in vivo, we designed a popliteal lymph node metastasis model in nude mice. A stable PRMT5 down‐regulation Tu686 cells with stably expressing firefly luciferase were inoculated into the footpads of nude mice. After six weeks, shPRMT5‐Luc cells effectively inhibited the metastasis to the popliteal lymph node through bioluminescence image scanning (Figure 2G, H). In addition, Western blot and IHC analysis showed that higher E‐cadherin, and lower PRMT5, MMP9, Vimentin levels in the PRMT5 knockdown group than in the control group (Figure 2I, J). Taken together, these findings validated that knockdown of PRMT5 suppresses laryngeal cancer cell invasion and lymph node metastasis in vivo.

### 3.3 PRMT5 promotes proliferation of laryngeal cancer cells in vitro and tumorigenesis in vivo

**FIGURE 3:**
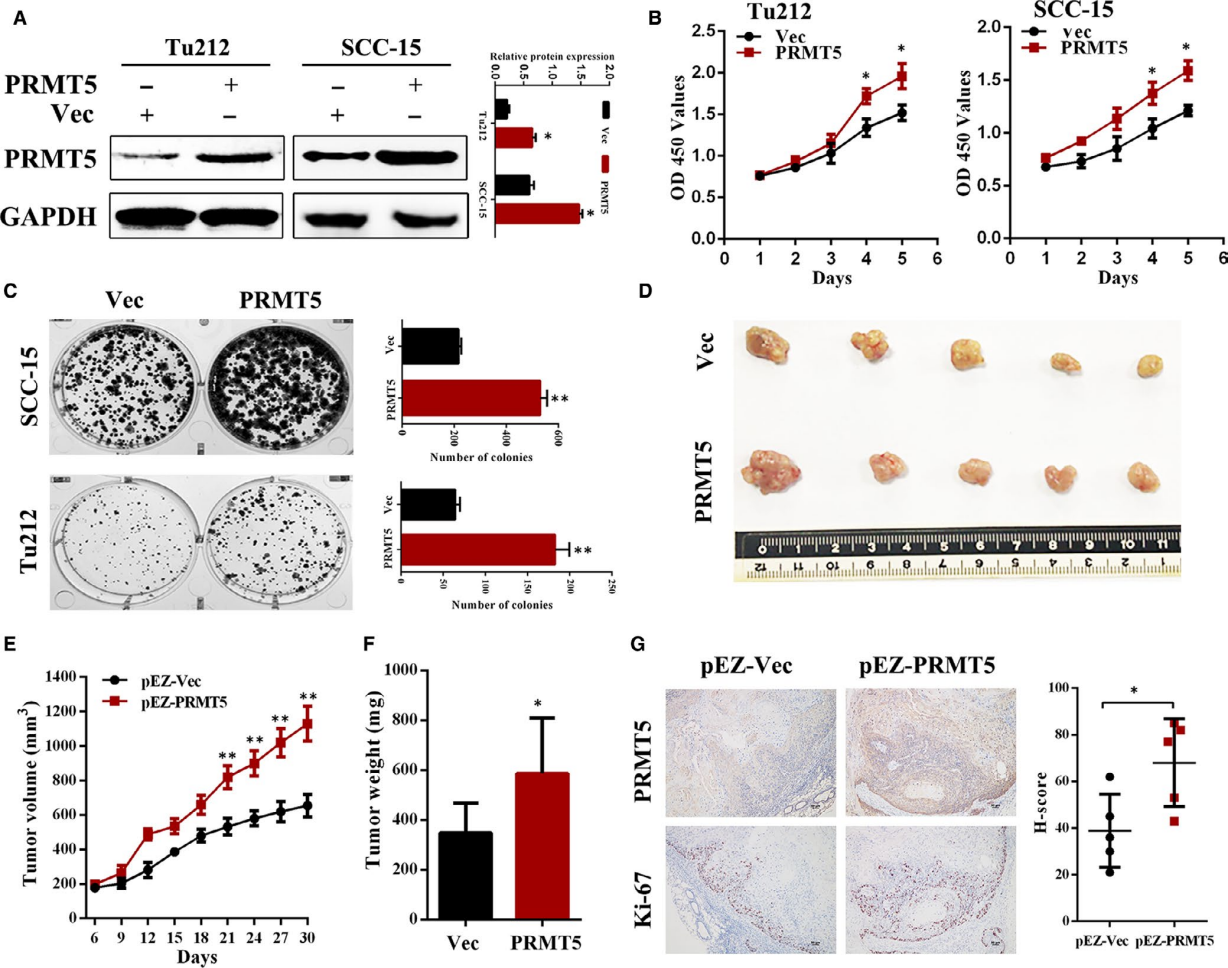
PRMT5 promotes proliferation of laryngeal cancer cells in vitro and tumorigenesis in vivo. A, Ectopic stable expression of PRMT5 in Tu212 and SCC‐15 cells compared to vector controls was confirmed by Western blot. B, Ectopic expression of PRMT5 significantly enhanced the proliferation of Tu212 and SCC‐15 cells shown by CCK8 assay. C, Colony formation assays were performed in PRMT5 overexpressing Tu212 and SCC‐15 cells. D, Representative images of the tumours of PRMT5 overexpression and control groups. E, Tumour growth curves were summarized in the line chart. F, Tumour weights were measured after the tumours were surgically dissected. G, IHC assay of PRMT5 and Ki‐67 in xenograft tumour tissues. The scale bar represents 50 μm. *P < .05, **P < .01

Gain‐of‐function study was performed by stably transfecting PRMT5 expression vectors into Tu212 and SCC‐15 cells. Ectopic expression of PRMT5 in these cells, as shown in Figure 3A, caused a significant increase in cell proliferation in both Tu212 and SCC‐15 cells compared to the empty vector transfected cells (Figure 3B). The colony formation assay suggested that PRMT5‐overexpressing cells increased the colony‐forming ability compared to cells with empty vector (Figure 3C). To further evaluate the effects of PRMT5 on laryngeal cancer cell tumorigenesis in vivo, stable PRMT5 overexpression cells (Tu212‐pEZ‐PRMT5) were implanted subcutaneously into nude mice and tumour growth was measured. Tumour size and weight were increased in PRMT5 overexpression group, compared with the corresponding group (Figure 3D‐F). Meanwhile, Tu212 xenografts overexpressing PRMT5 showed increased cell proliferation by Ki‐67 staining (Figure 3G). Collectively, these results suggest that PRMT5 promotes laryngeal cancer growth in vivo.

### 3.4 *PRMT5* is transcriptionally up‐regulated by YY1 in laryngeal cancer

**FIGURE 4:**
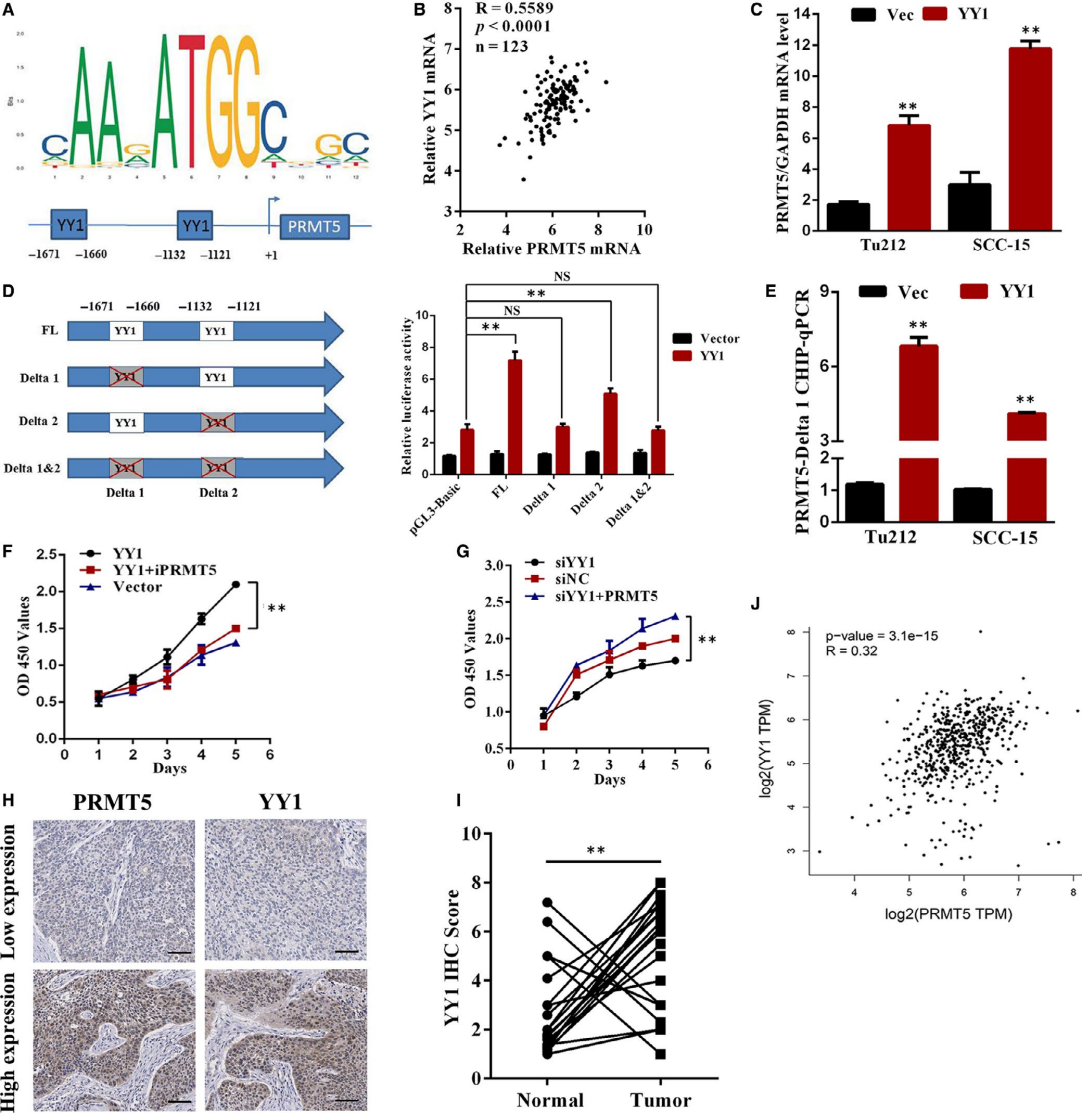
YY1 activated PRMT5 expression to promote the proliferation of laryngeal cancer cells. A, A schematic illustration of putative YY1 binding sites in the promoter of PRMT5. B, Dot plot showing YY1 and PRMT5 mRNA expression levels deduced from online data set: the TCGA larynx cancer data set (n = 123). C, The mRNA levels of PRMT5 were measured by qRT‐PCR in the indicated cells stably transfected with the vector or YY1 plasmid (mean ± SD, n = 3, **P < .01). D, Schematic illustration of PRMT5 promoter. Each square labelled represents a putative YY1 binding site (left panel). Tu212 cells with transfection of the pGL3‐PRMT5 promoter were co‐transfected with the vector or YY1 and subjected to dual‐luciferase reporter assays (right panel) (mean ± SD, n = 3, **P < .05, NS, no significant difference). E, ChIP real‐time PCR assays were conducted to assess the enrichment of YY1 to the *PRMT5* promoter region in Tu212 and SCC‐15 cells. Mean ± SD, **P < .01. F, PRMT5 inhibitor repressed YY1‐induced proliferation of Tu212 cells. **P < .01. G, The proliferation abilities inhibited by YY1 knockdown were recovered by up‐regulating PRMT5. **P < .01. H, Representative images show that patients with high expression of PRMT5 were frequently accompanied by high expression of YY1 protein. The scale bar represents 50 μm. I, Relative YY1 expression values were determined via IHC in 19 paired laryngeal cancer tissues. J, Dot plot showing the correlation between PRMT5 and YY1 expression deduced from online data set GEPIA

We next investigate the mechanism underlying transcriptional activation of PRMT5 in laryngeal cancer cells. As predicted by the JASPAR database,^37^ the potential binding sites for transcription factors were listed in Table S5. As the bioinformatic prediction, two potential YY1 binding sites (−1121 to −1132 and −1660 to −1671) were predicted in the promoter of *PRMT5* (Figure 4A). In addition, we found that PRMT5 mRNA was positively correlated with YY1 mRNA expression in the TCGA data set (Figure 4B). Therefore, we speculated that YY1 could transcriptionally up‐regulate PRMT5 in laryngeal cancer cells. To this end, as shown in Figure 4C, the mRNA levels of PRMT5 were increased when we overexpressed YY1 in both Tu212 and SCC‐15 cells compared to the control. In order to explore whether PRMT5 is a direct target of YY1, we constructed full length PRMT5 promoter activity vector, deletions of one or both YY1 binding elements in the dual‐luciferase reporter system in HEK293T cell. As illustrated in Figure 4D, we found that the mutant reporter with a fragment spanning from −1671 bp to −1660 bp binding sites depletion was crucial for the up‐regulation of the *PRMT5* promoter activity. Furthermore, Chromatin immunoprecipitation (ChIP) assay was used to verify whether YY1 binds directly to the *PRMT5* promoter (located at −1671~‐1660 bp) in Tu212 and SCC‐15 cells, as shown in Figure 4E, the ChIP results agreed with luciferase assay. In addition, whether YY1‐mediated PRMT5 up‐regulation contributed to the activation of laryngeal cancer cell proliferation had been determined. YY1 overexpression promoted the proliferation of laryngeal cancer cells, but was reversed by a PRMT5 inhibitor (GSK591) (Figure 4F). In contrast, the proliferation abilities inhibited by YY1 knockdown were most recovered by up‐regulating the expression of PRMT5 (Figure 4G).

In addition, IHC was performed to assess PRMT5 and YY1 expression in the context of laryngeal cancer tissues. The results indicated that YY1 was markedly increased in PRMT5‐positive laryngeal cancer tissues (Figure 4H). H‐scoring with 19 pairs of laryngeal cancer patient samples revealed significant up‐regulation of YY1 in laryngeal cancer tumour tissue compared to the adjacent normal tissue (Figure 4I). Strikingly, we found that the expression levels were positively correlated between YY1 and PRMT5 in laryngeal cancer tissues from GEPIA database (Figure 4J). Taken together, these data proved that YY1 directly and positively regulates PRMT5 transcription activity and cell proliferation in laryngeal cancer.

### 3.5 YY1/PRMT5 axis positively regulates EMT and cell migration in laryngeal cancer

**FIGURE 5:**
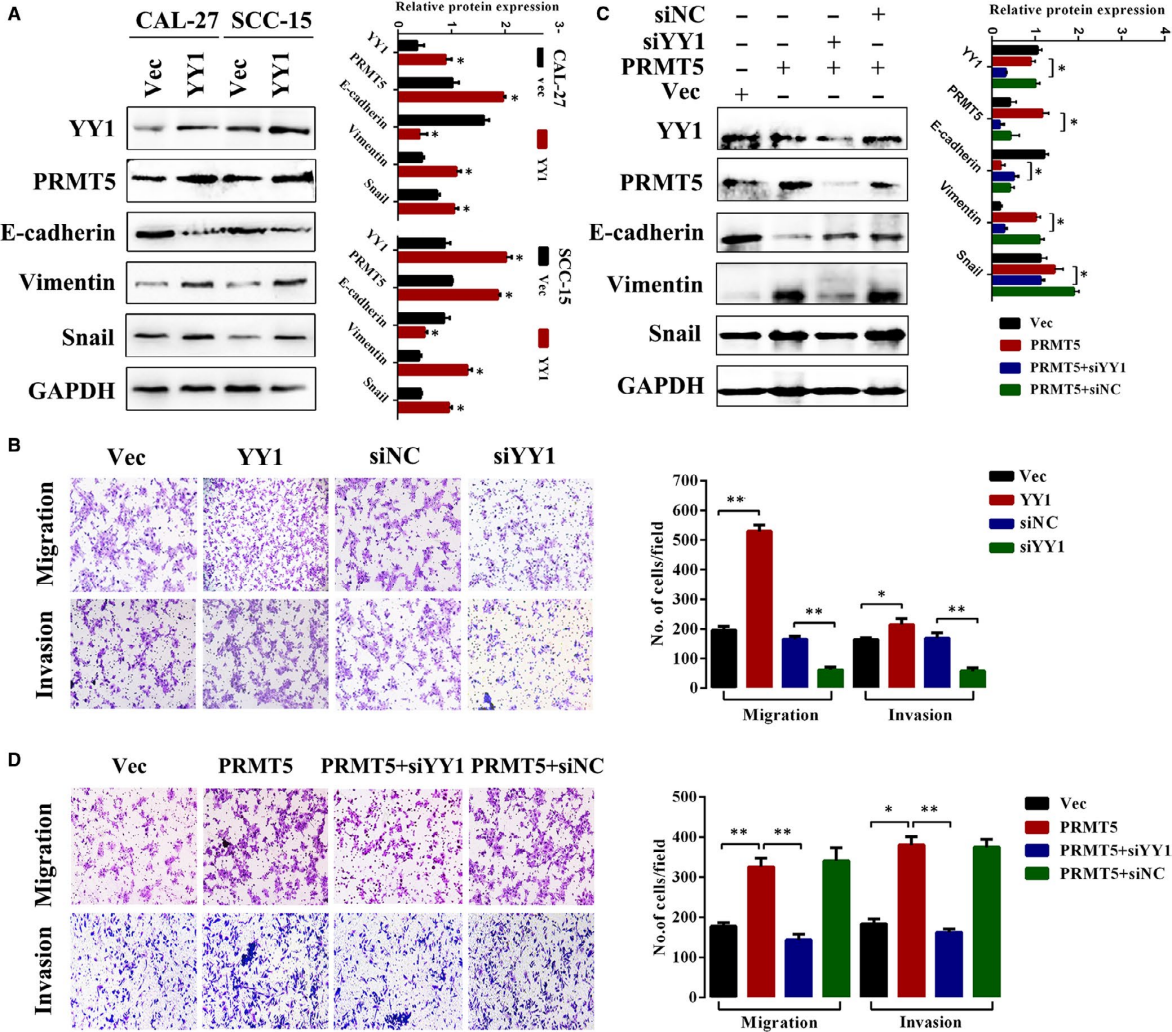
YY1/PRMT5 axis positively regulates EMT and cell migration in laryngeal cancer. A, CAL‐27 and SCC‐15 cells were transfected with YY1 or vector. Representative EMT‐related markers were immunoblotted. B, Cell migration and invasion were analysed using a transwell chamber or not. SCC‐15 and Tu212 cells with overexpression or knockdown of YY1 were placed on the upper chamber. Representative images shown in the left panel and quantitative data shown in the right panel. C, The effect of YY1 knockdown with PRMT5 overexpression on EMT marker expression in Tu686 cells. Representative images shown in left panel, and quantitative data shown in right panel. D, YY1 silencing inhibited PRMT5‐induced migration and invasion of laryngeal cancer cells. Representative images shown in the left panel, and quantitative data shown in the right panel. Each bar represents the mean ± SD (n = 3). *P < .05, **P < .01

Given that EMT is one of the hallmarks during cancer metastasis. Previous studies revealed that YY1 can act as a metastasis inducer and promote EMT in hepatocellular cancer.^27^ We next evaluated the YY1/PRMT5 axis signalling in the EMT regulation of laryngeal cancer. YY1 protein expression was analysed in laryngeal cancer cells (Figure S2). As shown in Figure 5A, overexpressed YY1 in CAL‐27 and SCC‐15 cells increased the protein levels of mesenchymal markers vimentin and snail but suppressed the epithelial marker E‐cadherin compared to the control. Meanwhile, transwell assays showed YY1 overexpression enhanced migration and invasion of laryngeal cancer cells, whereas the opposite outcome was observed after YY1 knockdown (Figure 5B). Furthermore, we explored whether YY1 is involved in the PRMT5‐induced EMT. As shown in Figure 5C, knockdown of YY1 in Tu686 cells increased PRMT5‐induced E‐cadherin reduction and reduced in vimentin and snail. Additionally, knockdown of YY1 reversed the PRMT5‐promoted cell migration and invasion of Tu212 cells (Figure 5D). This suggested that the YY1/PRMT5 axis enhances the metastatic potential in laryngeal cancer cells. Jointly, these results confirmed our previous hypothesis that the regulation of PRMT5 on EMT and metastasis depends on YY1.

**PRMT5 targets the Hippo signal**l**ing pathway to regulate laryngeal cancer metastasis.**

**FIGURE 6:**
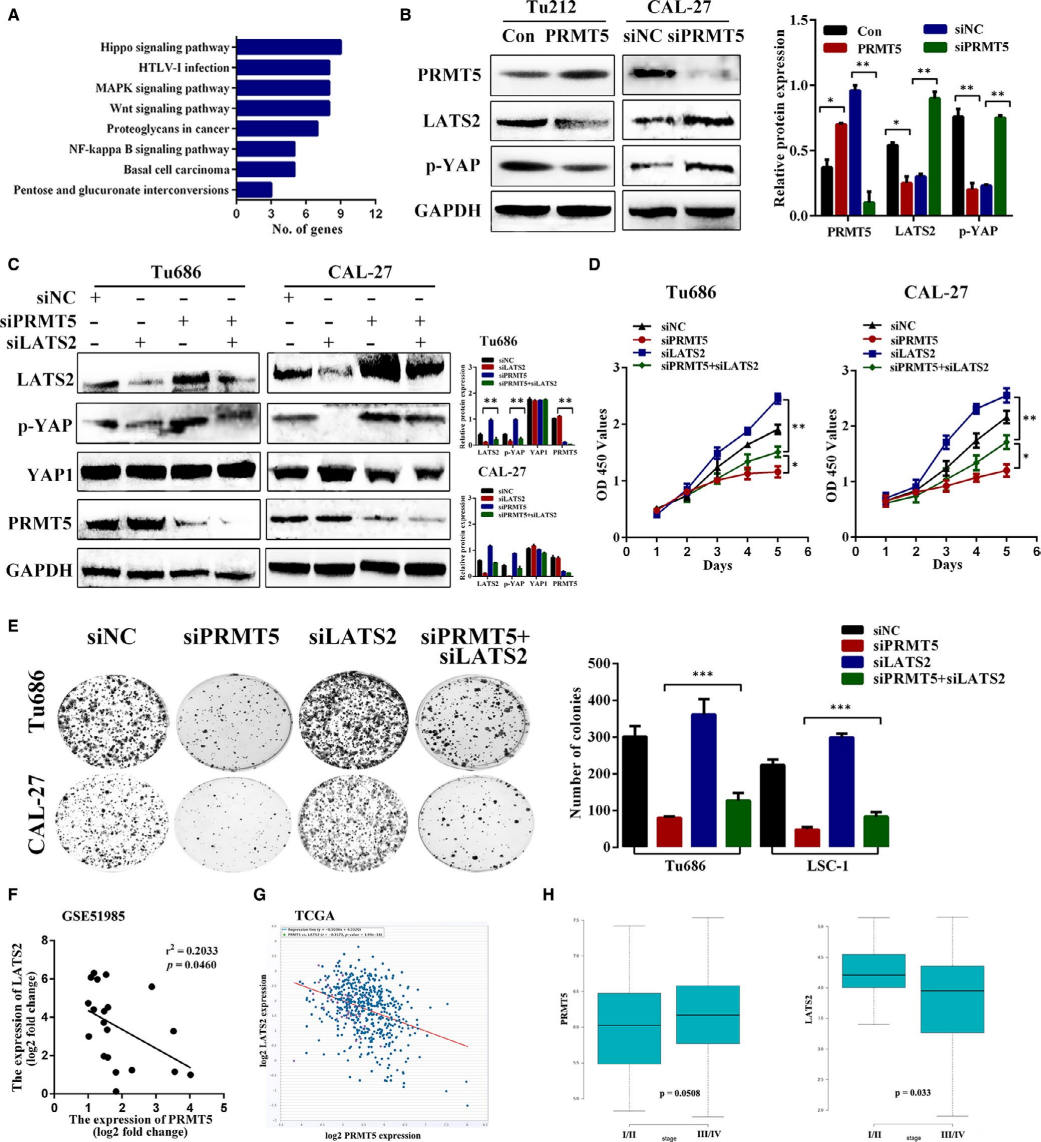
PRMT5 targets the Hippo signalling pathway to regulate laryngeal cancer metastasis. A, KEGG enrichment analysis showed the distribution of PRMT5‐regulated pathway. B, Western blot results showed that a lower level of LATS2 and p‐YAP1 in PRMT5 overexpression cells than that in the control group. A higher level of LATS2 and p‐YAP in siPRMT5 cells. C, Western blot of LATS2, YAP1, p‐YAP1 and PRMT5 expression upon LATS2 and PRMT5 double knockdown in laryngeal cancer cells. p‐YAP1 in LATS2 and PRMT5 double‐knockdown group was high in siLATS2 group, but lower than that of the siPRMT5 group. D, CCK8 assay showed siRNA‐mediated double knockdown of PRMT5/LATS2 inhibited cell growth compared to the siLATS2 group in Tu686 and CAL‐27 cells. E, Colony formation assay showed reduced cell colonies in siPRMT5/siLATS2 double‐knockdown cells, when compared to the siLATS2‐treated cells. Representative images shown in the left panel, and quantitative data shown in the right panel. F, GEO data set was downloaded for correlation analysis. G, PRMT5 and LATS2 expression from the TCGA head and neck cancer data set was analysed by the starBase database. H, Violin plots comparing mRNA expression levels of PRMT5 and LATS2 in TCGA head and neck cancer cohort. (mean ± SD, n = 3, *P < .05,**P < .01, ***P < .001)

To gain insights into the mechanism by which PRMT5 mediated EMT and metastasis, we performed RNA sequencing to identify the possible underlying signalling pathway of PRMT5 overexpressing. Kyoto Encyclopedia of Genes and Genomes (KEGG) pathway enrichment analysis showed that the Hippo signalling pathway was enriched upon PRMT5 overexpressed (Figure 6A). Meanwhile, Western blot was used to validate the Hippo pathway‐related factors LATS2 and p‐YAP expression upon PRMT5 overexpression /knockdown in Tu686 and CAL‐27 cells. Surprisingly, both of LATS2 and phos‐YAP were down‐regulated by PRMT5 overexpression and up‐regulated followed by PRMT5 knockdown, respectively (Figure 6B). To further investigate whether PRMT5 regulate laryngeal cancer metastasis by decreasing LATS2 expression, we transfected siLATS2 into Tu686 and CAL‐27 cells with siPRMT5 or siNC and evaluated the effect on phos‐YAP1 expression. We found that LAST2 depletion reduced phos‐YAP1 expression whereas no obvious alteration in the total YAP1 expression. Co‐transfection siPRMT5 and siLATS2 abolished the inhibitory effects of siLATS2 on phos‐YAP but could not fully restored the normal effect of siLATS2, suggesting that PRMT5 could partially rescue the effect of YAP1 dephosphorylation caused by siLATS2 (Figure 6C). Consistent with these results, we observed that double knockdown of PRMT5 and LATS2 by siRNA abolished the negative effects of siPRMT5 on cell proliferation and colony formation (Figure 6D,E). Furthermore, we performed correlation analysis of one laryngeal cancer GEO data set and found that *PRMT5* expression was negatively correlated with *LATS2* expression(Figure 6F). To further confirm this, we used the ChIPBase database and found the expression of PRMT5 was negatively associated with LATS2 expression (Figure 6G). Moreover, we found that PRMT5 expression was higher in more advanced stage, whereas LATS2 had the opposite result (Figure 6H). These findings suggested that the oncogenic role of PRMT5 is mediated through the LATS2/YAP down‐regulation in laryngeal cancer and this underlying mechanism was implicated in the regulation of laryngeal cancer metastasis.

## 4 DISCUSSION

Most cancer‐related deaths are caused by tumour metastasis.^38^ Although laryngeal cancer patients at early stage have a relatively better outcome, minimal improvement in survival of patients with metastasis illustrates the need to discover novel targets to explore new therapeutic approaches. By now, our understanding of the laryngeal cancer metastasis remains inadequate. Therefore, elucidation, the molecular mechanisms that drive tumorigenesis and/or metastasis of laryngeal cancer, is critical for clinical prevention. In this study, PRMT5 was found to be overexpressed and up‐regulated by YY1 in laryngeal cancer cells, in which it promoted metastasis by activating the LATS2 and YAP of hippo pathway. Moreover, patients with high PRMT5 expression predicted poor overall survival. We therefore propose that PRMT5 may be a potential target for treating metastatic laryngeal cancer.

PRMT5 is a member of the PRMTs family, which methylates arginines in both histones and non‐histone proteins.^39^, ^40^ In previous studies, PRMT5 has been determined as an oncoprotein in promoting the progression of various tumours, including mantle cell lymphoma, glioblastoma and leukaemias.^5^, ^41^, ^42^ In oral squamous cell carcinoma, PRMT5 acts as an EMT driver and may underlie the oncogene function of PRMT5 mediating cancer invasion and metastasis.^14^ Our study showed that PRMT5 was highly expressed in laryngeal cancer, and ectopic expression of PRMT5 promoted cell proliferation, migration and invasion of laryngeal cancer cells in vitro and facilitated tumour metastasis in vivo. In addition, lentivirus‐mediated down‐regulation of PRMT5 markedly inhibited cell proliferation and colony‐forming ability. More importantly, we confirmed that PRMT5 trigger EMT through LATS2 and YAP of Hippo signalling in laryngeal cancer cells. These results indicate new mechanistic of PRMT5 in laryngeal cancer‐invasion‐metastasis.

Transcription factors (TFs) are considered to play a key role in regulating gene expression. Abnormally expressing TFs are found in many types of cancers and gained increasing attention. YY1 itself is functionally diverse because it can act as an activator or inhibitor, which is based on the tumour type and its interacting partners.^22^ It has been reported that high expression of YY1 suppresses cell invasion and metastasis through down‐regulating MMP10 in pancreatic cancer,^28^ whereas aberrant YY1 expression contributes to cell cycle progression, cell proliferation, cell migration and invasion in melanoma.^43^ YY1 also facilitates cell movement by inducing EMT and contributes to cancer progression.^44^, ^45^ Here, we revealed that YY1 increases the proliferation, invasion and migration in laryngeal cancer cells, which is consistent with the oncogenic role of YY1 in laryngeal cancer.^46^ Our results showed that YY1 combined with the promoter of *PRMT5* and promoted the expression of PRMT5 to trigger EMT. In this YY1/PRMT5 axis, how do they bind to each other still needs further verification.

Poma *et al* indicated that metastasis of head and neck cancer related to the hippo pathway.^47^ YY1 has been reported as a metastasis‐related gene in pancreatic ductal adenocarcinoma.^27^ Therefore, we suggested whether YY1/PRMT5 axis has a connection with Hippo pathway. Our results discovered that YY1 enhanced laryngeal cancer invasion and migration, YY1/PRMT5 axis affect the protein level of LATS2 and phos‐YAP. We provide a novel notion that PRMT5 is transcriptionally up‐regulated by YY1 and decreases tumour repressor LATS2 expression thereby reduces the phosphorylation of YAP to enhance the invasive motility and promote EMT of laryngeal cancer cells. Therefore, one of our key findings demonstrated that PRMT5 as a novel regulator of the hippo pathway in laryngeal tumorigenesis. Also, our results seemed that siYY1 and PRMT5 co‐transfection even reduced the PRMT5 expression compared to controls. And overexpression of PRMT5 may up‐regulate YY1 expression, which is suggested that PRMT5 may also regulate YY1 as a feedback effects. The concrete mechanisms need to be further explored.

**FIGURE 7:**
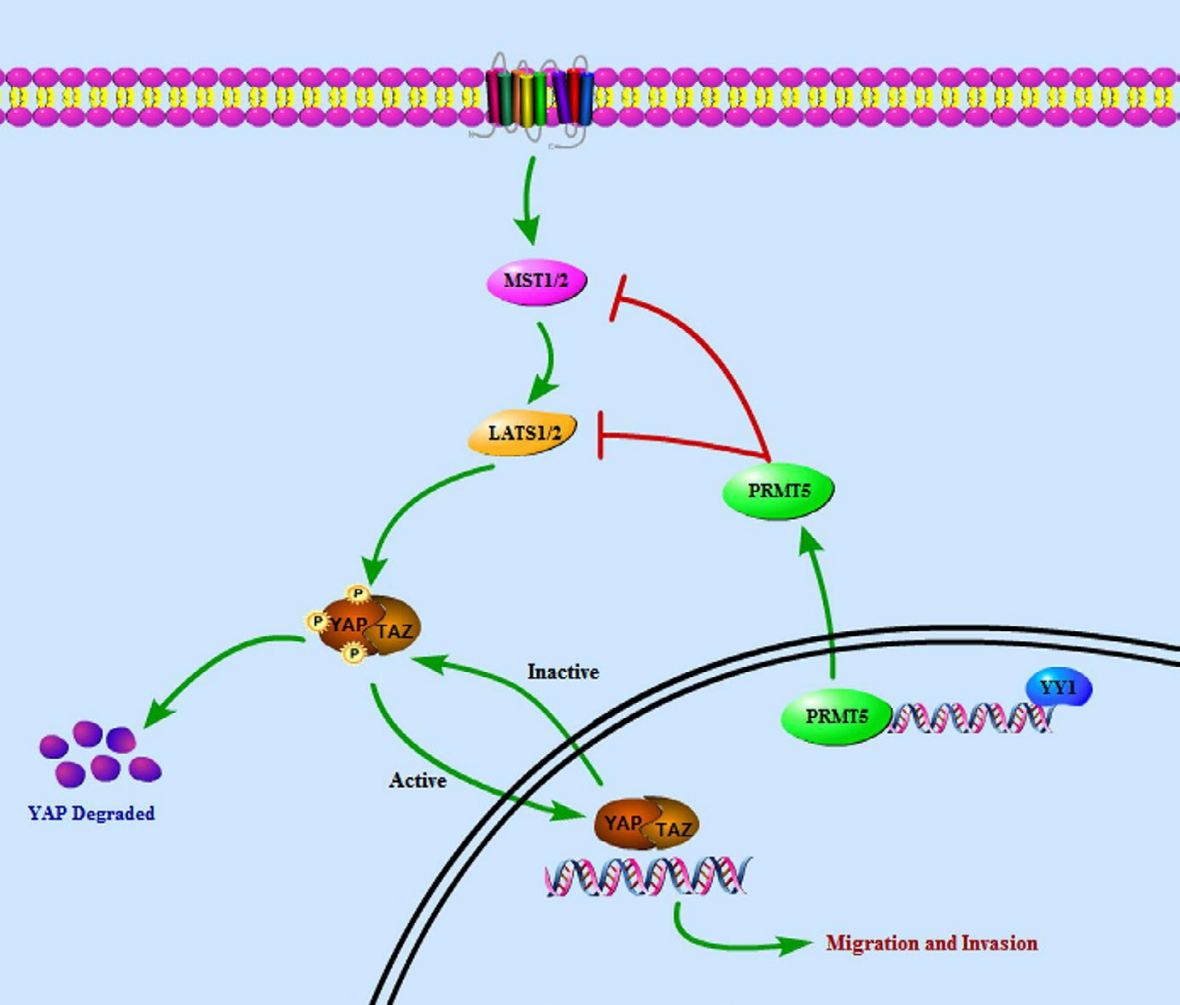
Proposed schematic mode of YY1/PRMT5/LATS2/YAP signalling axis in the pathogenesis of laryngeal cancer. YY1/PRMT5 axis is essential for Hippo pathway‐promoted proliferation and migration of laryngeal cancer cells

In summary, this study showed PRMT5 promoted tumour metastasis and correlated with a poor prognosis in laryngeal cancer. The underlying mechanism reveals YY1/PRMT5 axis enhances laryngeal cancer cell growth and invasive capacity via LATS2 and YAP signalling pathway (Figure 7). Therefore, inhibition of PRMT5 may serve as a promising therapeutic strategy for patients with lymphatic metastasis.

## CONFLICT OF INTEREST

The authors have declared that no competing interest exists.

## AUTHOR CONTRIBUTION

**Nan Wang:** Data curation (lead); Writing‐original draft (lead); Writing‐review & editing (lead). **Di Wu:** Formal analysis (equal); Writing‐original draft (equal). **Qian Long:** Formal analysis (supporting); Methodology (equal); Software (equal); Writing‐original draft (equal). **Yue Yan:** Formal analysis (supporting); Investigation (supporting); Software (supporting). **Xiaoqi Chen:** Methodology (equal); Software (supporting); Validation (equal). **Zheng Zhao:** Methodology (equal); Resources (equal); Validation (supporting). **Honghong Yan:** Validation (equal). **Xinrui Zhang:** Investigation (supporting); Software (equal); Validation (supporting). **Meilan Xu:** Formal analysis (equal); Investigation (equal); Validation (supporting). **Wuguo Deng:** Supervision (equal); Writing‐review & editing (equal). **Xuekui Liu:** Funding acquisition (lead); Project administration (equal); Supervision (equal); Writing‐original draft (supporting); Writing‐review & editing (equal).

## Supporting information

Figure S1

Figure S2

Table S1

Figure Legends

## Data Availability

Source data and reagents are available from the corresponding author upon reasonable request.
