# Supplementary figures and images for: Dysregulated YY1/PRMT5 axis promotes the progression and metastasis of laryngeal cancer by targeting Hippo pathway
Source: J Cell Mol Med. 2020 Dec 7;25(2):946–59. doi: 10.1111/jcmm.16156 (PMC7812261; doi:10.1111/jcmm.16156)

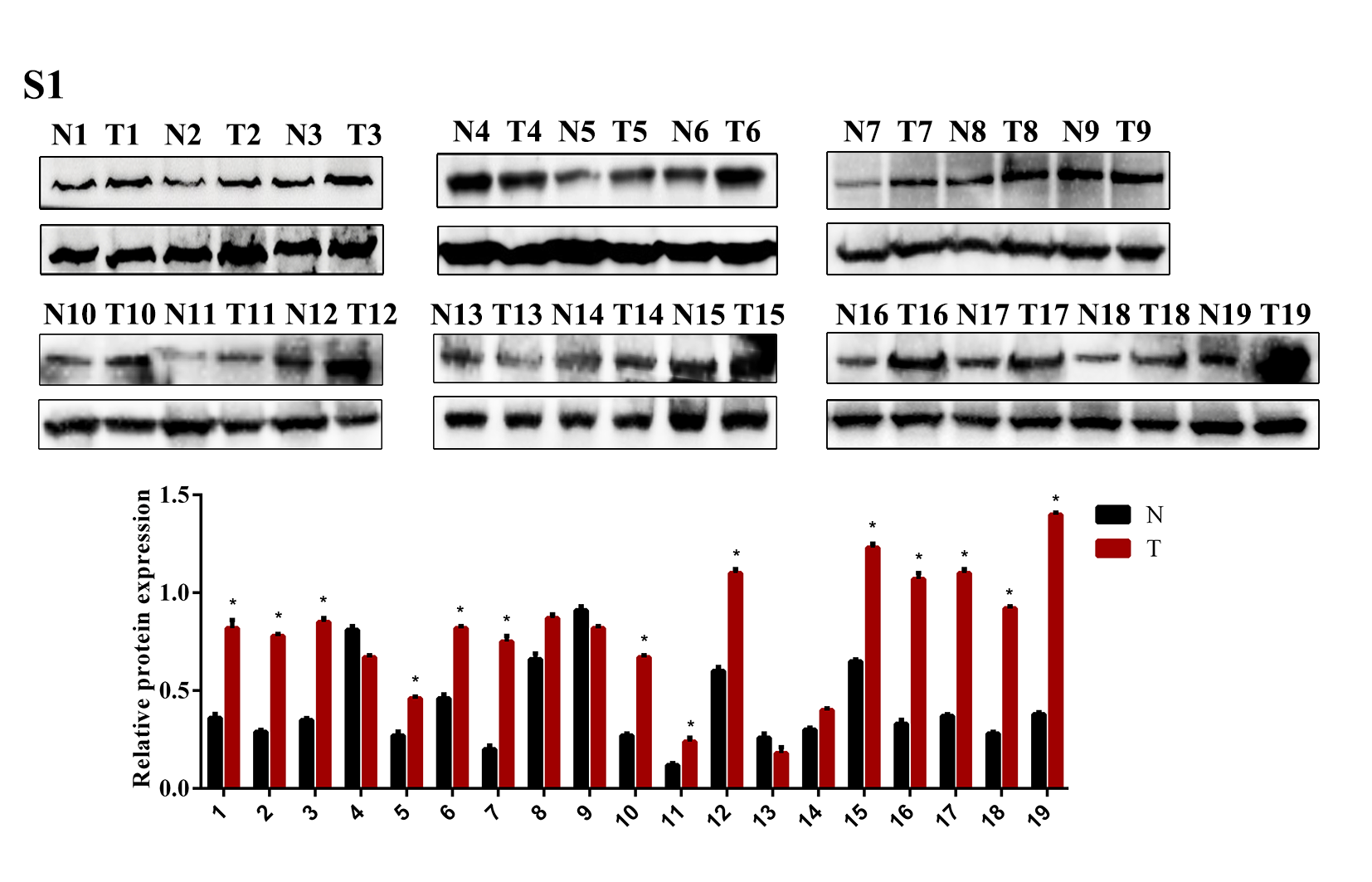

Supplement: Supplementary file 1 — Figure S1 [file JCMM-25-946-s001.tif]

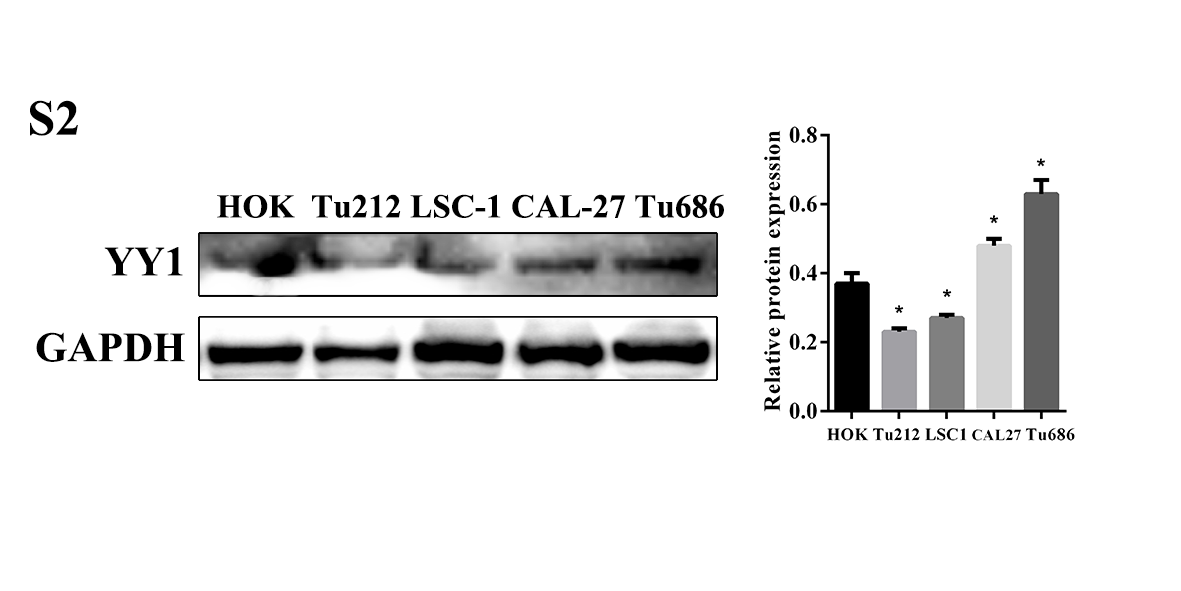

Supplement: Supplementary file 2 — Figure S2 [file JCMM-25-946-s002.tif]
