# Supplementary material for: Dysregulated YY1/PRMT5 axis promotes the progression and metastasis of laryngeal cancer by targeting Hippo pathway
Source: J Cell Mol Med. 2020 Dec 7;25(2):946–59. doi: 10.1111/jcmm.16156 (PMC7812261; doi:10.1111/jcmm.16156)
Supplement: Supplementary file 3 — Table S1 [file JCMM-25-946-s003.docx]

**Table S1. PRMT5-targeting shRNAs oligonucleotide sequences**

| shRNAs | Sequence (5’-3’) |
| --- | --- |
| sh1 forward: | 5’-CCGGGCACCAGTCTGTTCTGCTACTCGAGTAGCAGAACAGACTGGTGCTTTTTG-3’ |
| sh1 reverse: | 5’-AATTCAAAAAGCACCAGTCTGTTCTGCTACTCGAGTAGCAGAACAGACTGGTGC-3 |
| sh2 forward: | 5’-CCGGGAGGTGCAGTTCATCATCACTCGAGTGATGATGAACTGCACCTCTTTTTG-3 |
| sh2 reverse: | 5’-AATTCAAAAAGAGGTGCAGTTCATCATCACTCGAGTGATGATGAACTGCACCTC-3 |

**Table S2. The siRNA sequences used in this study**

| Name | Sequence (5’-3’) |
| --- | --- |
| PRMT5 | GCACCAGTCTGTTCTGCTA GAGGTGCAGTTCATCATCA |
| YY1 | CGACGACTACATTGAACAA CCTGAAATCTCACATCTTA |
| LATS2-2 | TCCGCAAAGGGTACACTCA |

**Table S3. DNA sequences of real-time PCR primers used for the detection**

**of mRNA expression.**

| Primer name | Sequence(5’-3’) |
| --- | --- |
| PRMT5 -F | 5’-CTGTCTTCCATCCGCGTTTCA-3’ |
| PRMT5 -R | 5’-GCAGTAGGTCTGATCGTGTCTG-3’ |
| YY1-F | 5’-ATACCTGGCATTGACCT-3’ |
| YY1-R | 5’-TGAGGGCAAGCTATTGT-3’ |
| GAPDH-F | 5′-TGTGGTCATGAGTCCTTCCA-3’ |
| GAPDH-R | 5’-CGAGATCCCTCCAAAATCAA-3’ |

**Table S4. Relationship between expression levels of PRMT5 in Laryngeal cancer**

**and clinicopathologic features**

| Characteristic | | No.  (n=80) | PRMT5 expression  High Low | *χ*^2^ | *p* |
| --- | --- | --- | --- | --- | --- |
|  |  |  |  |  |  |
| Age(years) |  | |  | 0.297 | 0.586 |
| <50 | | 24 | 11 13 |  |  |
| >50 | | 56 | 22 34 |  |  |
| Sex | |  |  | 0.025 | 0.874 |
| Male | | 68 | 38 30 |  |  |
| Female | | 12 | 7 5 |  |  |
| TNM stage | |  |  | 4.579 | **0.032^*^** |
| I II | | 53 | 33 20 |  |  |
| III IV | | 27 | 10 17 |  |  |
| Lymph node metastasis | |  |  | 5.657 | **0.017^*^** |
| No | | 70 | 35 35 |  |  |
| Yes | | 10 | 9 1 |  |  |
| Tumor size | |  |  | 0.008 | 0.929 |
| <2 cm | | 36 | 20 16 |  |  |
| >2 cm | | 44 | 24 20 |  |  |

**p*<0.05 values are set for significant differences

**Table S5. The potential binding sites for transcription factors**

| Model ID | Model name | | | Score | | Relative score | Start | End | | Strand | predicted site sequence |
| --- | --- | --- | --- | --- | --- | --- | --- | --- | --- | --- | --- |
| MA0095.2 | YY1 | | | 6.507 | | 0.819284853395614 | 1121 | 1132 | | 1 | TTACATGGCTGC |
| MA0095.2 | YY1 | | | 16.957 | | 0.970906469530549 | 1660 | 1671 | | 1 | AAAGATGGCGGC |
| MA0466.1 | | CEBPB | -1.770 | | 0.801385965258085 | | 56 | | 66 | -1 | GATCACGCCAC |
| MA0014.2 | | PAX5 | 6.587 | | 0.804515515515718 | | 64 | | 82 | -1 | GGTTGCAGTGAGCCAAGAT |
| MA0014.2 | | PAX5 | 7.339 | | 0.81330137111635 | | 208 | | 226 | -1 | GAGGTCAGGAGTTCGAGAC |
| MA0466.1 | | CEBPB | 0.028 | | 0.820232854221018 | | 246 | | 256 | 1 | GCTTTCCAAAG |
| MA0014.2 | | PAX5 | 6.699 | | 0.805824047200919 | | 280 | | 298 | -1 | AAAACCGGCCGGGCGCGGT |
| MA0518.1 | | Stat4 | 6.222 | | 0.833574328270043 | | 337 | | 350 | -1 | GTTCTGGGGAACCC |
| MA0466.1 | | CEBPB | 2.080 | | 0.841742206919537 | | 465 | | 475 | 1 | TATTACACAGC |
| MA0466.1 | | CEBPB | -1.372 | | 0.805557857253217 | | 499 | | 509 | -1 | GGTTTGGTCAT |
| MA0466.1 | | CEBPB | 1.515 | | 0.83581979742896 | | 518 | | 528 | 1 | GATTTCCCAAA |
| MA0466.1 | | CEBPB | 0.216 | | 0.822203496670979 | | 544 | | 554 | 1 | TCTTGCTCACT |
| MA0014.2 | | PAX5 | 7.055 | | 0.809983308628877 | | 743 | | 761 | -1 | GAAGGAAGCAAGGAAAGAC |
| MA0466.1 | | CEBPB | 2.746 | | 0.848723312619934 | | 826 | | 836 | 1 | AATTTCATGAC |
| MA0518.1 | | Stat4 | 13.504 | | 0.936017506720721 | | 936 | | 949 | -1 | ATTCTAGGAAGTAG |
| MA0466.1 | | CEBPB | 0.406 | | 0.824195103402324 | | 986 | | 996 | -1 | TGTTACATATT |
| MA0466.1 | | CEBPB | -0.307 | | 0.816721337089437 | | 1021 | | 1031 | 1 | AATAGCACCAG |
| MA0466.1 | | CEBPB | -0.705 | | 0.812549445094305 | | 1073 | | 1083 | -1 | TCTTGTGTCAT |
| MA0518.1 | | Stat4 | 12.569 | | 0.922863926406057 | | 1102 | | 1115 | 1 | GTTACGGGAAAAGC |
| MA0466.1 | | CEBPB | 3.317 | | 0.854708614954658 | | 1127 | | 1137 | 1 | GGCTGCATAAC |
| MA0014.2 | | PAX5 | 7.268 | | 0.812471855494482 | | 1154 | | 1172 | 1 | CTGATAATGAAAGCGTGAA |
| MA0466.1 | | CEBPB | 1.216 | | 0.832685637362266 | | 1157 | | 1167 | -1 | GCTTTCATTAT |
| MA0466.1 | | CEBPB | 8.861 | | 0.91282160294715 | | 1210 | | 1220 | 1 | AATTACAAAAT |
| MA0518.1 | | Stat4 | 7.278 | | 0.848430136625428 | | 1507 | | 1520 | 1 | TTGGCAGGAAAAGC |
| MA0466.1 | | CEBPB | 0.476 | | 0.824928853250714 | | 1724 | | 1734 | 1 | AATTGCGTCCC |
