# Supplementary material for: Dysregulated YY1/PRMT5 axis promotes the progression and metastasis of laryngeal cancer by targeting Hippo pathway
Source: J Cell Mol Med. 2020 Dec 7;25(2):946–59. doi: 10.1111/jcmm.16156 (PMC7812261; doi:10.1111/jcmm.16156)
Supplement: Supplementary file 4 — Figure Legends [file JCMM-25-946-s004.docx]

**Supplementary figure 1.** Western blot analysis of PRMT5 expression in nineteen laryngeal cancer tissues and matched non-tumorous tissues.Bar plot showing relative

protein PRMT5 expression levels in individual patients (1–19).

**Supplementary figure 2.** The expression of YY1 in different head and neck cancer cells.
